# microRNA as a Potential Vector for the Propagation of Robustness in Protein Expression and Oscillatory Dynamics within a ceRNA Network

**DOI:** 10.1371/journal.pone.0083372

**Published:** 2013-12-23

**Authors:** Claude Gérard, Béla Novák

**Affiliations:** Oxford Centre for Integrative Systems Biology, Department of Biochemistry, University of Oxford, Oxford, United Kingdom; Sun Yat-sen University, China

## Abstract

microRNAs (miRNAs) are small noncoding RNAs that are important post-transcriptional regulators of gene expression. miRNAs can induce thresholds in protein synthesis. Such thresholds in protein output can be also achieved by oligomerization of transcription factors (TF) for the control of gene expression. First, we propose a minimal model for protein expression regulated by miRNA and by oligomerization of TF. We show that miRNA and oligomerization of TF generate a buffer, which increases the robustness of protein output towards molecular noise as well as towards random variation of kinetics parameters. Next, we extend the model by considering that the same miRNA can bind to multiple messenger RNAs, which accounts for the dynamics of a minimal competing endogenous RNAs (ceRNAs) network. The model shows that, through common miRNA regulation, TF can control the expression of all proteins formed by the ceRNA network, even if it drives the expression of only one gene in the network. The model further suggests that the threshold in protein synthesis mediated by the oligomerization of TF can be propagated to the other genes, which can increase the robustness of the expression of all genes in such ceRNA network. Furthermore, we show that a miRNA could increase the time delay of a “Goodwin-like” oscillator model, which may favor the occurrence of oscillations of large amplitude. This result predicts important roles of miRNAs in the control of the molecular mechanisms leading to the emergence of biological rhythms. Moreover, a model for the latter oscillator embedded in a ceRNA network indicates that the oscillatory behavior can be propagated, via the shared miRNA, to all proteins formed by such ceRNA network. Thus, by means of computational models, we show that miRNAs could act as vectors allowing the propagation of robustness in protein synthesis as well as oscillatory behaviors within ceRNA networks.

## Introduction

MicroRNAs (miRNAs) are short noncoding RNA molecules of 20–30 nucleotides, which can bind to the 3′ UTR of messenger RNA resulting in a post-transcriptional repression of protein synthesis by targeting the corresponding messenger RNA for degradation and/or by inhibiting its translation [1], [2].

Besides the key role of miRNAs for the down-regulation of protein expression [3]–[5], miRNAs can induce thresholds in protein synthesis [6]. Moreover, they are often involved in feed-forward regulations with their target genes, allowing an increase in the robustness of protein expression towards molecular noise [6]–[11]. Such robustness may be also achieved by protein oligomerization of transcription factors (TF) for the control of genetic expression [12].

Moreover, miRNAs have been also found to be critical regulators in many molecular regulatory networks driving animal development and human diseases [13]. Indeed, miRNAs could act as oncogenes or tumor suppressors and are able to control critical steps in cancer development [14]–[20], such as the process of cell transformation [21] and the epithelial-to-mesenchymal transition (EMT transition) [22]–[24]. The latter transition is crucial for the initiation and the development of metastasis. miRNAs are also involved in molecular regulatory networks driving cell differentiation in multiple tissues [15], [25]–[27].

Furthermore, the presence of miRNAs as well as oligomerization of TF for the control of gene expression has been suggested to play key roles in the regulation of the ordered temporal pattern of proteins expression during developmental processes [11], [28]–[30].

Without resorting to any feedback or feed-forward regulation between a miRNA and its target gene, we will propose here minimal models for protein expression to study the effect of miRNA as well as oligomerization of TF on protein synthesis. By resorting to stochastic simulations, we will assess the effect of miRNAs and oligomerization of TF on the robustness of protein expression towards molecular noise. The robustness of protein synthesis will be also analyzed in a deterministic model for a heterogeneous cell population, which accounts for the dynamical variation that may arise between cells in a population.

Previous studies indicate that different competing endogenous RNAs (ceRNAs) may be interconnected via their binding to a common miRNA, which can lead to the co-regulation of the various proteins expressed by such ceRNA network [31], [32]. The correct balance in the activity of ceRNAs and miRNAs seems important for the development of physiological and pathological conditions, such as in cancer [31], [33]. Such balance may be deregulated since ceRNAs can act as miRNA sponges [31], [34]. Based on those studies, we will also assess the effect of multiple messenger RNAs (ceRNAs) linked to the same miRNA on the dynamics of protein synthesis. We will analyze the effect of oligomerization of TF for the control of gene expression on the dynamics of such ceRNA network.

Since miRNAs could be also important regulators in the molecular mechanisms leading to the occurrence of biological rhythms [35], [36], we will further study the effect of miRNA on the dynamical behavior of a “Goodwin-like” oscillator [37]. The latter oscillator has been extensively used to model the molecular dynamics driving the circadian clock in diverse organisms [38]–[40]. Finally, we will propose a model to account for the potential dynamics of a “Goodwin-like” oscillator embedded in a ceRNA network.

## Results

### Minimal model for the synthesis of one protein regulated by a miRNA

**Figure 1:**
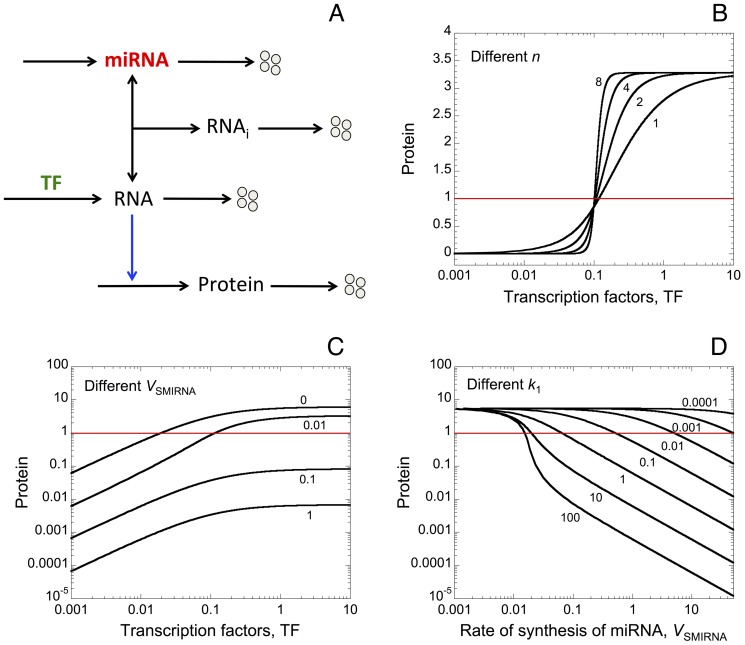
Oligomerization of transcription factors (TF) and miRNAs induce thresholds in protein expression. (A) Minimal model for protein synthesis including a miRNA. TF activate, with or without oligomerization of TF, the synthesis of a messenger RNA (RNA). This RNA can form an inhibitory complex, RNA_i_, with a miRNA, which prevents it to encode the synthesis of the protein. We assume that the level of each component in the model can be controlled by synthesis and degradation. Steady-state levels of protein *vs* the level of TF are shown for different degrees of oligomerization of TF (n) in B, and for different rates of synthesis of miRNA (*V*_SMIRNA_) in C. Horizontal red lines indicate an arbitrary threshold of protein needed to promote a cellular response. (D) Steady-state levels of protein *vs V*_SMIRNA_ for different rate constants of association between the RNA and the miRNA, *k*_1_. Parameter values used in the simulations are as in Table 2.

First we propose a minimal model for protein expression to account for the effect of miRNA as well as oligomerization of TF on protein output (see wiring diagram in Fig. 1A). In order to assess only the effect of miRNA and oligomerization of TF on protein synthesis, the model does not incorporate any assumptions about feedback and feed-forward regulations between the miRNA and its target gene [41]. Moreover, we do not include complex molecular regulatory mechanisms that may arise to control the temporal and spatial patterns of gene expression [42]. Here, TF activate, with or without cooperativity mediated by a certain degree of oligomerization, the synthesis of messenger RNA. The binding of miRNA to the RNA forms an inhibitory complex RNA_i_, which prevents the messenger RNA to encode the synthesis of the protein. The model is based on 4 kinetic equations describing the time evolution of miRNA, RNA_i_, RNA, and protein (see section: “Methods”). The different variables are defined in Table 1, while Table 2 gives a definition, together with their numerical values, of the different parameters of the model.

**Table 1:** Variables of the model.

Symbol	Definition
RNA	Messenger RNA
Prot	Protein encoded by the messenger RNA
RNA_i_	Inhibitory complex between RNA and miRNA
miRNA	microRNA, which forms inhibitory complexes with the corresponding messenger RNAs
**Addition of a two messenger RNAs, RNA2 and RNA3, which can also bind to miRNA**	
RNA2	Messenger RNA 2
Prot2	Protein encoded by the messenger RNA 2
RNA2_i_	Inhibitory complex between RNA2 and miRNA
RNA3	Messenger RNA 3
Prot3	Protein encoded by the messenger RNA 3
RNA3_i_	Inhibitory complex between RNA3 and miRNA
**“Goodwin-like” oscillator with miRNA regulation of protein synthesis**	
Rep	Repressor, which can prevent the synthesis of the messenger RNA

**Table 2:** Parameters of the models.

Symbol	Definition	Numerical value
**1) Minimal model for protein synthesis with a messenger RNA regulated by a miRNA**		
*V*_SRNA_	Rate of synthesis of messenger RNA	0.02
*TF*	Transcription factors	
*K*_ARNA_	Michaelis constant for activation of RNA synthesis by TF	0.1
*k*_1_	Bimolecular rate constant for binding of miRNA to RNA	10
*k*_2_	Rate constant for dissociation of complex (RNA_i_) between miRNA and RNA	0.01
*k*_DRNA_	Rate constant for the degradation of RNA	0.1
*k*_SPROT_	Rate constant for the synthesis of protein	3
*k*_DPROT_	Rate constant for the degradation of protein	0.1
*k*_DRNAI_	Rate constant for the degradation of RNA_i_	0.1
*V*_SMIRNA_	Rate of synthesis of miRNA	
*k*_DMIRNA_	Rate constant for the degradation of miRNA	0.1
*n*	Coefficient of cooperativity mediated by the oligomerization of TF needed to promote the transcription of messenger RNA	
**2) Minimal model with two messenger RNAs regulated by the same miRNA**(Other parameter values are as in section 1 of this Table)		
*V*_SRNA_	Rate of synthesis of messenger RNA	3
*V*_SRNA2_	Rate of synthesis of messenger RNA 2, RNA2	0.02
*V*_SRNA3_	Rate of synthesis of messenger RNA 3, RNA3	0.02
*k*_3_	Bimolecular rate constant for binding of miRNA to RNA2	10
*k*_4_	Rate constant for dissociation of complex (RNA2_i_) between miRNA and RNA2	0.01
*k*_5_	Bimolecular rate constant for binding of miRNA to RNA3	10
*k*_6_	Rate constant for dissociation of complex (RNA3_i_) between miRNA and RNA3	0.01
*k*_DRNA2_	Rate constant for the degradation of RNA2	0.1
*k*_DRNA3_	Rate constant for the degradation of RNA3	0.1
*k*_SPROT2_	Rate constant for the synthesis of protein 2, Prot2	3
*k*_SPROT3_	Rate constant for the synthesis of protein 3, Prot3	3.5
*k*_DPROT2_	Rate constant for the degradation of Prot2	0.1
*k*_DPROT3_	Rate constant for the degradation of Prot3	0.1
*k*_DRNAI2_	Rate constant for the degradation of RNA2_i_	0.1
*k*_DRNAI3_	Rate constant for the degradation of RNA3_i_	0.1
**3) “Goodwin-like” oscillator with regulation of protein synthesis by a miRNA**		
*V*_SMIRNA_	Rate of synthesis of miRNA	0.1
*k*_DMIRNA_	Rate constant for the degradation of miRNA	0.01
*k*_1_	Bimolecular rate constant for binding of miRNA to RNA	100
*k*_2_	Rate constant for dissociation of complex (RNA_i_) between miRNA and RNA	0.01
*V*_SRNA_	Rate of synthesis of messenger RNA	0.4
*k*_DRNA_	Rate constant for the degradation of RNA	0.4
*k*_SPROT_	Rate constant for the synthesis of protein	2
*k*_DPROT_	Rate constant for the degradation of protein	0.02
*k*_DRNAI_	Rate constant for the degradation of RNA_i_	0.1
*k*_7_	Rate constant for the conversion of the protein into the repressor (i.e. transport of the protein from the cytosol to the nucleus and/or post-translational modifications)	0.1
*k*_8_	Rate constant for the conversion of the repressor into the protein (i.e. transport of the repressor from the nucleus to the cytosol and/or post-translational modifications)	0.01
*K*_IMRNA_	Constant of inhibition by the repressor of the synthesis of RNA	0.04
*k*_DREP_	Rate constant for the degradation of repressor, Rep	0.3
*n*	Coefficient of oligomerization between repressor molecules to inhibit the synthesis of messenger RNA	12

**Notes:** The minimal models proposed here could represent the generic dynamics of protein expression in various types of eukaryotic cells. Such dynamics may vary in an extensive manner for different types of proteins within a cell and may also vary between different cell types. Thus, in order to stay as general as possible, we have chosen a set of ‘representative’ dimensionless parameter values.

As expected, the model shows that an increase in the coefficient of oligomerization of TF, n, generates a sharp threshold in the expression of the protein (see Fig. 1B as well as [43]). Steady-state levels of protein towards TF for different rates of synthesis of miRNA, *V*_SMIRNA_, indicate that an increase in the level of miRNA reduces the overall level of protein output (Fig. 1C). Regardless of the level of TF, the model shows that a sufficient amount of miRNA, i.e for *V*_SMIRNA_ = 0.1 or 1, can completely prevent the occurrence of a cellular response, which is defined by an arbitrary threshold of protein (see horizontal red lines in Fig. 1). Furthermore, steady-state levels of protein *vs V*_SMIRNA_ for different rate constants of association between the messenger RNA and the miRNA, *k*_1_, show that the presence of miRNA can induce threshold in protein output (Fig. 1D). The latter result confirms previous experimental as well as theoretical observations showing the occurrence of thresholds in protein synthesis generated by the presence of miRNA [6].

#### 1. miRNA and oligomerization of TF as robust buffers towards stochastic fluctuations in protein synthesis

**Figure 2:**
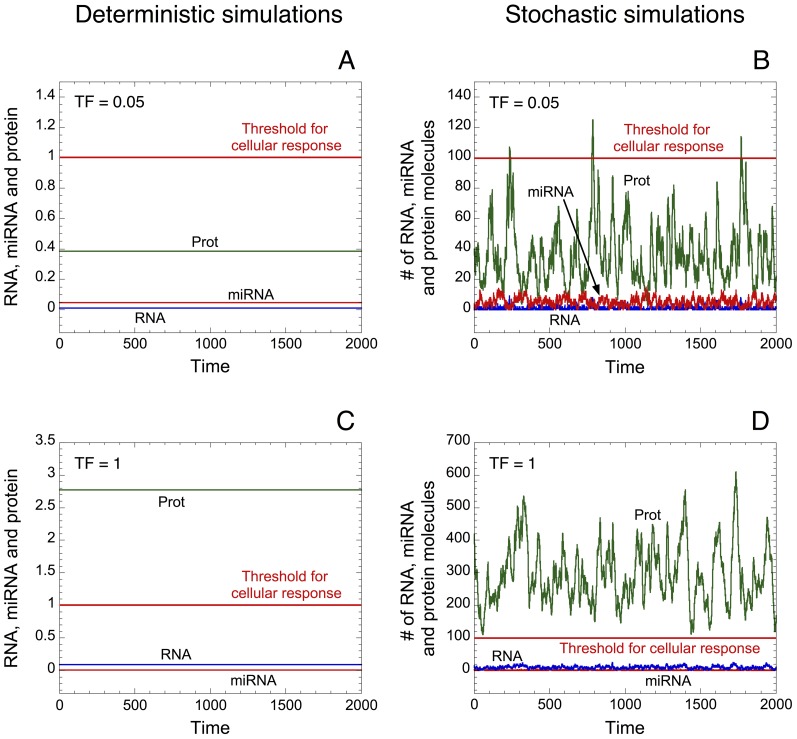
Effect of stochastic fluctuations on protein expression. Deterministic (A, C) and the corresponding stochastic (B, D) time evolution of RNA, protein and miRNA are shown in the presence of low (TF = 0.05 in A, B) or high levels of TF (TF = 1 in C, D). The level of protein is low with small levels of TF and large with high levels of TF (compare panels A and C). Here again, horizontal red lines define an arbitrary threshold of protein needed to elicit a cellular response. Small stochastic fluctuations in the copy number of messenger RNA molecules can greatly affect the level of protein output (see panels B and D). Stochastic simulations were performed by means of the Gillespie's algorithm [87] using the stochastic version of the minimal model for protein synthesis (see Table 3). The units on the axes for the stochastic curves are expressed in numbers of molecules. The corresponding concentrations for the deterministic trajectories are obtained by dividing the numbers of molecules by Ω expressed in units of 10^6^ L/N_A_, where N_A_ is Avogadro's number. Here, as well as in Figs. 3 and S1, Ω = 100. The rate of synthesis of miRNA, *V*_SMIRNA_, is equal to 0.01; the coefficient of cooperativity, *n*, is equal to 1; and the other parameter values are as in Table 2.

Protein synthesis can be strongly influenced by stochastic process [44], [45]; such stochasticity can greatly influence the level of protein output [46]. A stochastic version of the deterministic model proposed in Fig. 1A supports those results by showing that small random variations in the copy number of messenger RNA can result in large fluctuations in the protein level (see Fig. 2 as well as Table 3 for the kinetic equations used in the stochastic version of the model). Indeed, the deterministic (Fig. 2A, C) and the corresponding stochastic time evolution of RNA, miRNA and protein (Fig. 2B, D) illustrated with low (TF = 0.05 in Fig. 2A, B) or high levels of TF (TF = 1 in Fig. 2C, D) indicate that stochastic fluctuations can greatly influence the dynamics of protein output. Moreover, if we set an arbitrary threshold of proteins above which a cellular response is elicited (see horizontal red lines in Fig. 2), stochastic simulations indicate that an abusive cellular response could be generated due to molecular noise even in the presence of low levels of TF (see Fig. 2B).

**Table 3:** Stochastic version of the model (see scheme in Fig. 1A).

Reaction number	Reaction	Propensity of reaction
1	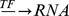	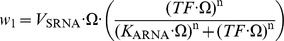
2	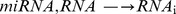	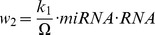
3	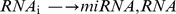	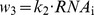
4	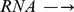	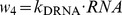
5	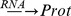	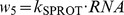
6	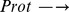	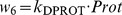
7	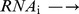	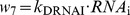
8	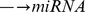	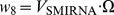
9	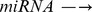	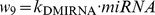

**Figure 3:**
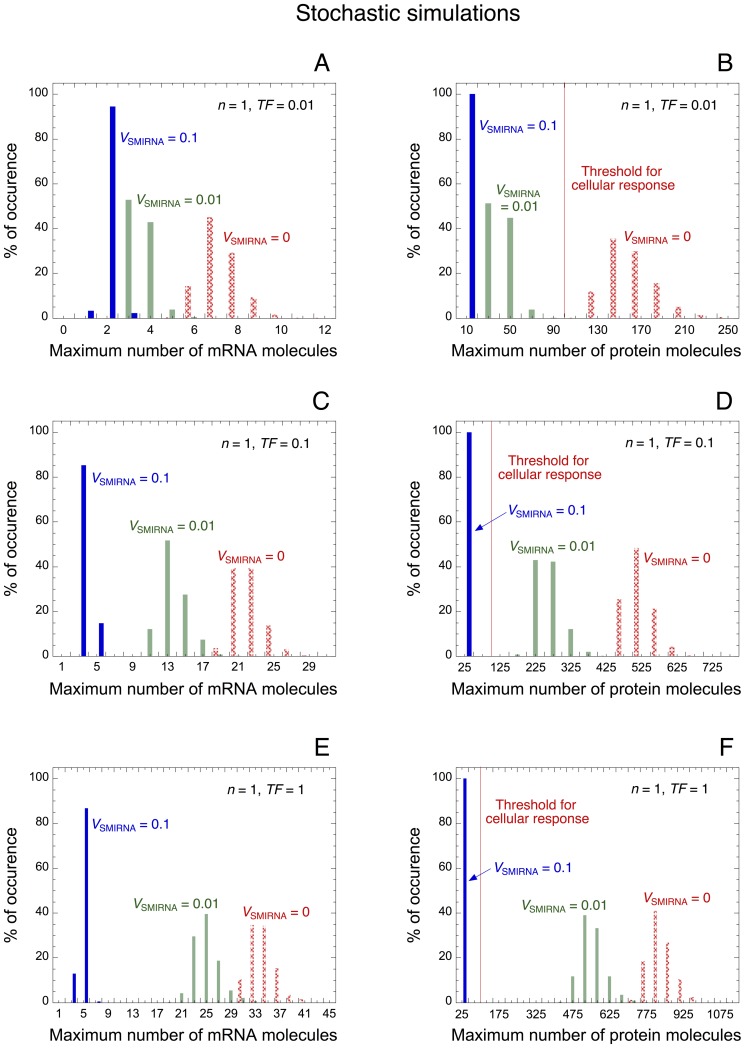
miRNA as a robust buffer for the synthesis of protein. Stochastic distribution of the maximum number of messenger RNA and protein molecules in the presence of low (TF = 0.01 in A and B), intermediate (TF = 0.1 in C and D), and large levels of TF (TF = 1 in E and F). For each case, the distribution is calculated with 1000 stochastic cells (see stochastic model for protein expression in Table 3) in the absence of miRNA (*V*_SMIRNA_ = 0) as well as in the presence of low and high levels of miRNA (*V*_SMIRNA_ = 0.01 and 0.1). Increasing the level of miRNA reduces the overall level of messenger RNA and protein. Vertical red lines in panels B, D and F indicate the arbitrary threshold of protein (100 protein molecules) needed to promote a cellular response. Regardless of the level of TF, a high level of miRNA, *V*_SMIRNA_ = 0.1, ensures a robust repression of protein synthesis, which impedes the occurrence of a cellular response; while with low levels of TF, a small level of miRNA, *V*_SMIRNA_ = 0.01, is already sufficient to prevent stochastic occurrence of protein expression (see panel B). Parameter values are as in Table 2.

By using the stochastic version of the minimal model for protein expression, we can assess the effect of miRNA on the control and the robustness of protein expression. The distribution of maximum number of messenger RNA and protein molecules is represented for low (TF = 0.01 in Fig. 3A, B), intermediate (TF = 0.1 in Fig. 3C, D), and high levels of TF (TF = 1 in Fig. 3E, F). For each condition, different rates of synthesis of miRNA, *V*_SMIRNA_, are considered. As in the deterministic case, the stochastic model indicates that an increase in the level of miRNA reduces the overall level of messenger RNA and protein. Furthermore, the model shows that high levels of miRNA, *V*_SMIRNA_ = 0.1, can generate a robust buffer towards stochastic fluctuations. Indeed, regardless of the level of TF, with high levels of miRNA the protein output never exceeds the arbitrary threshold, i.e. 100 protein molecules, needed to ensure a cellular response (see Fig. 3B, D, F). In the presence of low level of TF, the model also indicates that a small level of miRNA, *V*_SMIRNA_ = 0.01, is already sufficient to create a robust buffer towards undesirable stochastic expression of the protein (Fig. 3B).

The model indicates that the copy number of messenger RNAs (less than 40 molecules per cell) and proteins (hundreds of molecules per cell) is in the same order of magnitude as observed experimentally. Indeed, quantitative measurements of messenger RNA and protein molecules in *E. coli* [47], in yeast [48], as well as in mammals [49] show an expression of messenger RNA of about tens of copies per cell, while the expression of proteins is in the range of hundreds or thousands of copies per cell.

The maximum number of protein molecules as a function of TF represented in the absence (Fig. S1A, C) or in the presence of miRNA (Fig. S1B, D), as well as with (Fig. S1C, D) or without (Fig. S1A, B) oligomerization of TF indicates that miRNA and oligomerization of TF generate a robust buffer towards stochastic fluctuations. Indeed, with low levels of TF, this buffer prevents the stochastic occurrence of supra-threshold amounts of protein. The latter result stresses the fact that oligomerization of TF for the control of gene expression represents a very good molecular mechanism to create a buffer in protein expression (compare panels C and D with panels A and B in Fig. S1). As mentioned above, such robustness of protein synthesis mediated by oligomerization of TF and/or by miRNA seems to be crucial to ensure the control of an ordered deterministic pattern of protein expression required during developmental processes [11], [29]. Mechanisms leading to noise minimization may be particularly important for some classes of genes, such as essential genes and those involved in complex-forming proteins [50]. Recently, it was shown that gene network architecture is also an important regulator controlling the variability of gene expression. In particular, a network of interlocked positive and negative feedback loops can be an effective mechanism to ensure developmental robustness [51]. However, note also that in some circumstances, stochastic gene expression can be advantageous by providing the flexibility required to adapt to fluctuating environments or to respond to sudden stresses [52].

#### 2. Dynamics of protein synthesis in a heterogeneous cell population

In the previous section, we analyzed the effect of intrinsic molecular noise on the dynamics of protein expression. Here, we propose a model to account for the dynamics of protein synthesis in a heterogeneous cell population, where parameter values for each cell can vary in a random manner. Such parameter variation could represent the dynamical heterogeneity that may arise between cells in a population [53]–[55]. Based on this model, we will assess the effect of miRNA on the dynamics of protein expression in a cell population.

**Figure 4:**
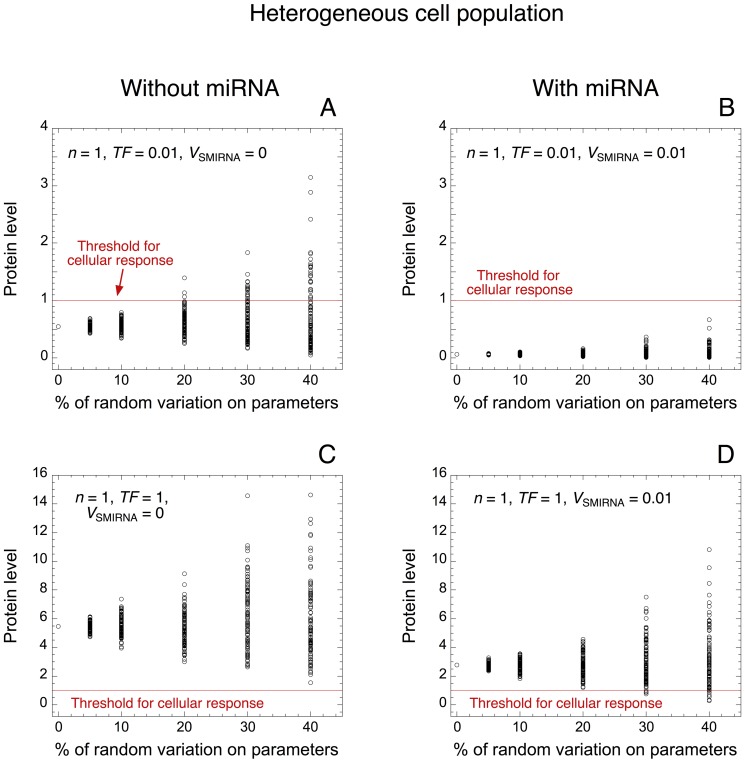
Robustness of protein expression towards random variation of parameter values. Levels of protein are shown towards the percentage of random variation around the default value for every parameter of the model in the absence (*V*_SMIRNA_ = 0 in A, C) or presence of miRNA (*V*_SMIRNA_ = 0.01 in B, D), as well as with low (TF = 0.01 in A, B) or high levels of TF (TF = 1 in C, D). This simulation may represent the dynamics of protein synthesis in a heterogeneous cell population. Red lines indicate the arbitrary threshold of protein needed to ensure a cellular response. With a low level of TF, miRNA robustly inhibits the undesirable expression of protein, even when 40% of random variation is considered on each parameter of the model (compare panels A and B). For each condition, 100 deterministic cells with random variation on each parameter are considered. Each dot corresponds to the protein level of one cell. Default values for the parameter are as in Table 2. For all simulations involving heterogeneous cell population, the parameter variation is applied with a uniform distribution around the mean value for each parameter.

Protein levels are represented towards the percentage of random variation for each parameter of the model in the absence (*V*_SMIRNA_ = 0 in Fig. 4A, C) or in the presence of miRNA (*V*_SMIRNA_ = 0.01 in Fig. 4B, D), as well as in the presence of low (TF = 0.01 in Fig. 4A, B) or high levels of TF (TF = 1 in Fig. 4C, D). Without random variation from the default value of the parameters, the model shows that, with low levels of TF, the protein level is small (below the threshold needed to generate of cellular response) (see Fig. 4A, B), while the protein level is above the threshold with high levels of TF (Fig. 4C, D). By increasing the random variation from 0 to 40% around the default value for each parameter, the model indicates that, in the absence of miRNA and in the presence of low levels of TF, a significant proportion of cells abusively exhibit a cellular response (see Fig. 4A). The presence of miRNA creates a robust buffer that prevents those undesirable cellular responses (Fig. 4B). In the presence of high levels of TF, a cellular response is always generated (Fig. 4C). In the latter condition, the model suggests that with large random variations around the default value of the parameters, the addition of miRNA might in few cases prevent the occurrence of the desired cellular response (see Fig. 4D). However, this observation happens only for few cells and with a large random variation (30% or 40%).

### Model for the expression of proteins formed by a minimal ceRNA network

**Figure 5:**
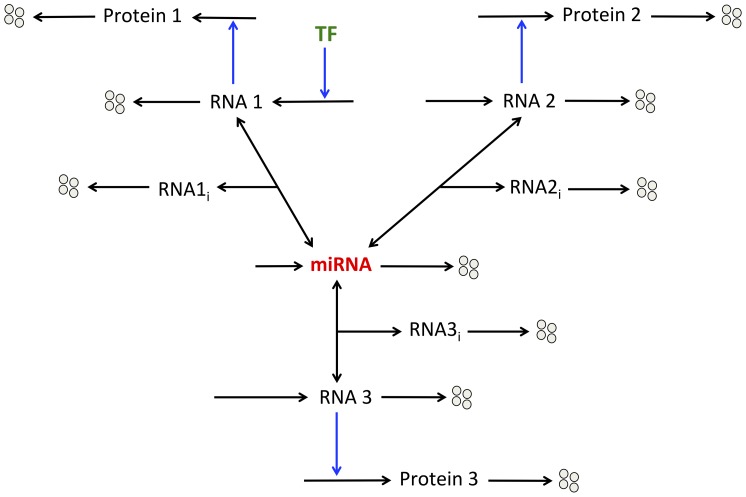
Model for the dynamics of a minimal ceRNA network. The expression of three proteins (Proteins 1, 2, and 3) is controlled by the same miRNA. Transcription factors (TF) only promote, with or without cooperativity mediated by their oligomerization, the synthesis of messenger RNA 1. The different messenger RNAs (RNAs 1, 2, 3) can form inhibitory complexes (RNA1_i_, RNA2_i_, RNA3_i_) with the miRNA, which prevent them to encode the synthesis of their respective protein.

Here, we extend the previous model by considering that the same miRNA binds and inhibits three messenger RNAs: RNA 1, 2 and 3 (see wiring diagram in Fig. 5). Those RNAs are ceRNAs for each other. TF only activate, with or without oligomerization of TF, the synthesis of RNA 1. We do not consider regulation of the expression of RNA 2 or RNA 3 by TF. The three RNAs encode the synthesis of their respective proteins.

The model counts 10 kinetic equations describing the time evolution of the different variables of the model (see section 2 in Methods: “Minimal model: 3 ceRNAs regulated by the same miRNA” as well as Table 1 for a definition of the different variables of the model).

**Figure 6:**
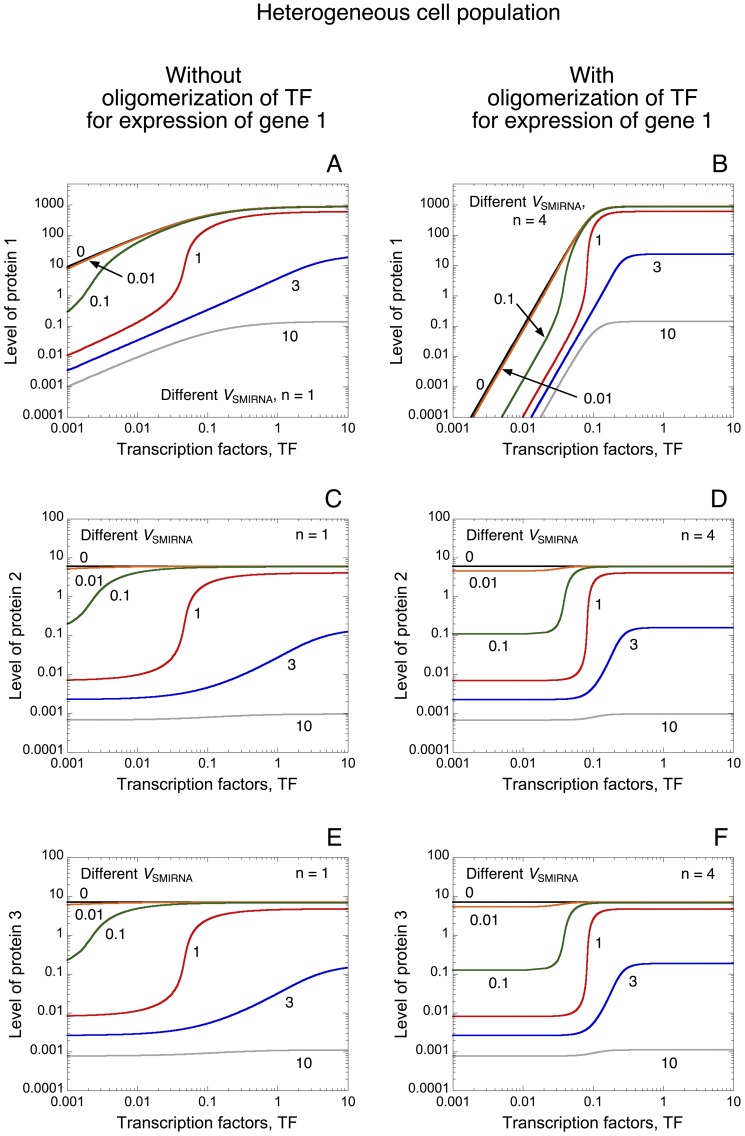
Propagation of thresholds in protein synthesis within a ceRNA network. Steady-state levels of protein 1 (A, B), protein 2 (C, D), and protein 3 (E, F) *vs* TF are shown in the absence (n = 1 in A, C, E) or in the presence of oligomerization of TF for the expression of gene 1 (n = 4 in B, D, F). For each case, different rates of synthesis of miRNA, *V*_SMIRNA_, are considered: *V*_SMIRNA_ = 0, 0.01, 0.1, 1, 3 and 10. For low levels of TF, the level of protein 1 is greatly reduced when oligomerization of TF increases from n = 1 to 4 (compare panels A and B). As a result of common miRNA regulation, TF can control the levels of proteins 2 and 3 and can induce thresholds in the expression level of those proteins (compare panel C with D and panel E with F). Parameter values are as in Table 2.

Steady-state levels of protein 1 towards the level of TF established in the absence or presence of oligomerization of TF indicates, as expected, that oligomerization of TF induces an abrupt switch in protein expression below which the gene is repressed and above which it is expressed (see Fig. 6A and 6B as well as Fig. 1). In each case, the rise in the level of miRNA reduces the overall level of protein. Even if TF only control the synthesis of RNA 1, as a result of the shared miRNA between the three messenger RNAs, the model indicates that TF can also regulate the steady-state level of proteins 2 and 3 (Fig. 6C–F). Indeed, a positive correlation in the expression pattern of the three proteins is observed. Such positive correlations between a gene and its pseudogene, i.e. between the phosphatase PTEN and its pseudogene pten1, as well as between a messenger RNA and its ceRNA have already been observed theoretically and experimentally [31], [32], [56]–[59]. Obviously, in the absence of miRNA, the level of TF does not influence the level of proteins 2 and 3 (see Fig. 6C–F for *V*_SMIRNA_ = 0).

Interestingly, the model predicts that, via the presence of miRNA, the oligomerization of TF allowing the occurrence of an abrupt switch in the expression pattern of protein 1 can be propagated to the expression profiles of proteins 2 and 3 (compare Fig. 6C with Fig. 6D as well as Fig. 6E with Fig. 6F showing the level of protein 2 and protein 3 towards TF in the absence or presence of oligomerization of TF, respectively). This result suggests that, with adequate level of miRNA, the expression of all proteins involved in a ceRNA network can be completely repressed in the absence of TF regulating the expression of only one gene in this network, while all proteins can be expressed in the presence of such TF.

Since the model suggests that thresholds in protein expression mediated by oligomerization of TF can be propagated within a ceRNA network, could it be possible that the robustness of gene expression mediated by such thresholds be transmitted to the whole network?

**Figure 7:**
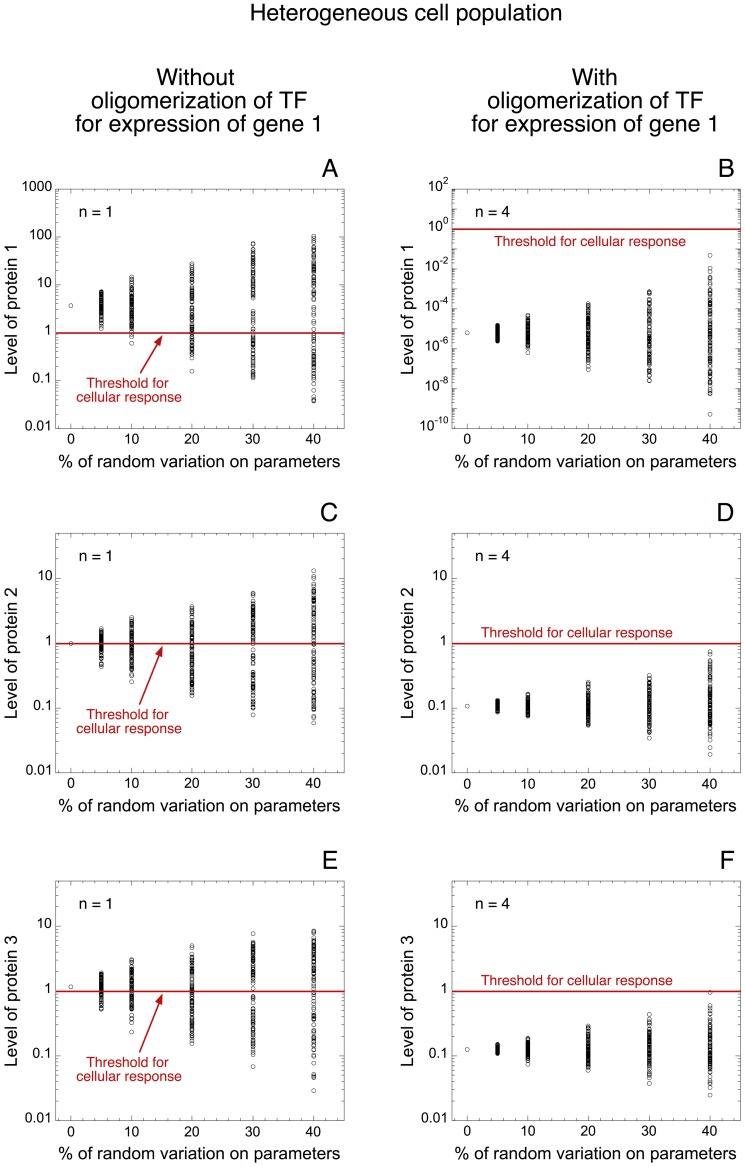
Propagation of robustness in protein expression within a ceRNA network. Levels of protein 1 (A, B), protein 2 (C, D) and protein 3 (E, F) are shown in a heterogeneous cell population as a function of the percentage of random variation around the default value for each parameter of the model. Simulations are performed without (n = 1 in A, C, E) and with oligomerization of TF for the synthesis of gene 1 (n = 4 in B, D, F). Red lines indicate the arbitrary threshold of protein needed to generate a cellular response. As a result of common miRNA regulation, the buffer effect in protein 1 expression, mediated by the oligomerization of TF, is propagated to the expression profile of proteins 2 and 3 (compare panels C with D and panels E with F). In each case, a low level of TF is considered (TF = 0.0025) and the rate of synthesis of miRNA, *V*_SMIRNA_, is equal to 0.1 (see green curve in Fig. 6 with TF = 0.0025). Simulations are performed with 100 deterministic cells for each condition. Default values for the parameters are as in Table 2.

To answer that question, we resort to a model for a ceRNA network in a heterogeneous cell population (see Fig. 5). We represent the level of proteins 1, 2, and 3 towards the percentage of random variation on every parameter of the model. In all cases, a low level of TF is considered (TF = 0.0025 in Fig. 7). This low level of TF is still sufficient to ensure a cellular response in the absence of oligomerization of TF (see levels of proteins 1, 2 and 3 in Fig. 7A, C, E without random variation on parameters). The model indicates that the presence of oligomerization of TF for the expression of gene 1 drastically decreases the level of all proteins (compare Figs. 7A, C, E with Figs. 7B, D, and F, respectively). Moreover, the model suggests that a large proportion of cells will exceed the threshold in the absence of oligomerization (Fig. 7A, C, E), while all proteins are well repressed in the presence of oligomerization, even with 40% of random variation on each parameter of the model (Fig. 7B, D, F).

Thus, the model indicates that the threshold in protein synthesis elicited by the oligomerization of TF for the expression of one gene can be propagated, via a shared miRNA, to the pattern of expression of the different genes involved in such ceRNA network. If insufficient levels of TF are present, the propagation of this threshold elicits a robust buffer for the whole ceRNA network preventing the expression of the other genes. While all proteins in the network can be expressed with supra-threshold amounts of TF (Fig. 6B, D, F for high levels of TF).

However, the model suggests that the propagation of such threshold may only occur if adequate levels of miRNA are present. Indeed, an elevated level of ceRNA can sponge the free available miRNA (Fig. S2A where steady-state levels of protein 2 are shown towards the level of TF for different rates of synthesis of RNA 3, *V*_SRNA3_). The rise in the level of RNA 3 (ceRNA) sponges the available miRNA, which impedes the propagation of thresholds in protein synthesis in this minimal ceRNA network (Fig. S2A). The model also predicts that an elevated level of miRNA can counteract, to some extent, this effect (see Fig. S2B when the rate of synthesis of miRNA, *V*_SMIRNA_ is equal to 1 instead of 0.1 in the conditions of Fig. S2A).

In the simulations of Figs. 6, 7, and S2, the kinetics of expression of genes 2 and 3 is very similar. To analyze the effect of different kinetics of expression between ceRNAs on the robustness of protein output within a ceRNA network, we consider different kinetics of expression for genes 2 and 3 (see Figs. S3 and S4). In one set of parameter values, set 2, the kinetics of expression of gene 3 is much faster than the kinetics of expression of gene 2, while the opposite is applied in set 3 (see legend of Fig. S3). The model indicates that when the kinetics of expression of one gene in the network is much larger that the others, the latter can sponge the available miRNA (see the steady-state levels of miRNA in the different conditions of Fig. S3). The reduction in the level of free miRNA (compare Fig. S3B with Figs. S3A and S3C) causes an increase in the levels of proteins 2 and 3. Such increase prevents, in the framework of a heterogeneous cell population, the existence of a robust buffer of protein output within the ceRNA network (compare Fig. S4A and S4C with Fig. S4B and S4D).

The latter result supports a recent theoretical study showing that the interaction of miRNAs with their different target messenger RNAs, which forms a ceRNA network, can be described by a titration mechanism [59]. The latter mechanism is characterized by threshold effect defined by the amount of shared miRNAs and messenger RNA targets. At the proximity of the threshold, a maximum correlation between messenger RNA targets is observed, as well as the presence of robustness of ceRNA effect with respect to random parameter variation [59]. In our model, because TF can regulate the transcription of one gene in the network, it could also move the latter threshold (defined by the relative levels of messenger RNAs and miRNA) to different amounts of miRNA. Furthermore, another recent theoretical study compared the mechanisms of eukaryotic microRNA-regulated genes with the regulation of gene expression by the prokaryotic small non-coding RNAs (sRNAs) [60]. Interestingly, they showed that, despite different mechanisms of regulation, the expression levels and the noise profiles of regulated-genes are almost identical in eukaryotes and prokaryotes.

Thus, a proper balance in the levels of ceRNAs and miRNAs is critical for the control of the activity of a ceRNA network. Such ceRNA network activity may be an important layer of regulation for physiological and pathological developments [32], [33].

### miRNA as a source of time delay for biological oscillators

It has been shown that miRNAs may be also important modulators of the negative regulatory circuits allowing sustained oscillatory behaviors in many physiological systems. Those physiological systems lead to the occurrence of biological rhythms [61], such as the NFκB oscillations [62]; Hes1 (Notch signaling effector) ultradian oscillations [63]; the calcium oscillations [35]; the cell cycle [64]–[67]; as well as the circadian clock [36], [68]–[72].

**Figure 8:**
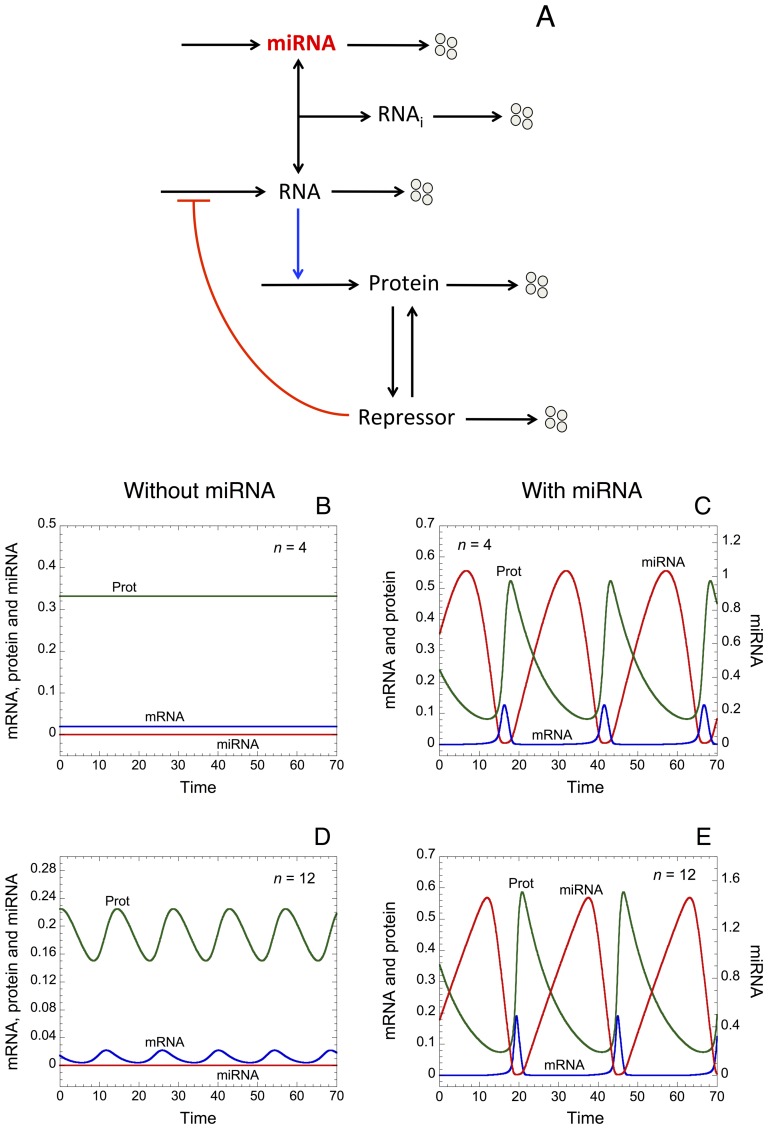
“Goodwin-like” oscillator including a miRNA. (A) Wiring diagram of the oscillator. A messenger RNA encodes the synthesis of a protein, which can convert into a repressor, i.e. due to post-translational modifications of the protein. The repressor impedes the synthesis of the messenger RNA, which creates a negative feedback loop allowing sustained oscillations in the levels of RNA, protein, and repressor. Here, a miRNA can regulate the level of RNA by forming an inhibitory complex, RNA_i_. Time evolution of RNA, miRNA and protein is shown in the absence (*V*_SMIRNA_ = 0 in B, D) or in the presence of miRNA (*V*_SMIRNA_ = 0.1 in C, E); as well as in the presence of intermediate (*n* = 4 in B, C) or high degree of oligomerization (*n* = 12 in D, E) amongst repressor molecules to down-regulate the synthesis of RNA. The model shows that the presence of miRNA promotes sustained oscillations of large amplitude. Parameter values are as in Table 2.

Here, by resorting to a minimal “Goodwin-like” oscillator model (see [37], [38] as well as Fig. 8A); we will assess the effect of a miRNA on the dynamics of such oscillator. The model rests on a negative feedback loop where a messenger RNA encodes the synthesis of a protein, which can be converted into a repressor. The latter represses the synthesis of the messenger RNA. By forming an inhibitory complex with the messenger RNA, the miRNA inhibits the expression of the protein.

The “Goodwin-like” oscillator model proposed here counts 5 kinetic equations (see Eqs. 11 to 15 in Methods) describing the time evolution of the different variables of the model (see also Table 1 for a definition of the different variables).

Time evolution of messenger RNA, protein and miRNA in the absence (*V*_SMIRNA_ = 0 in Fig. 8B and 8D) or in the presence of miRNA (*V*_SMIRNA_ = 0.1 in Fig. 8C and 8E), as well as in the presence of intermediate (n = 4 in Fig. 8B and 8C) or high levels of cooperativity in repression for the down-regulation of gene expression (n = 12 in Fig. 8D and 8E) indicates that oligomerization of repressors and miRNA promote the occurrence of sustained oscillatory behavior. Regardless of the degree of oligomerization, the model also predicts that, at least with the parameter values used, oscillations of only small amplitude are observed without miRNA (Fig. 8D), while large amplitude of oscillations are elicited in its presence (Fig. 8C and 8E).

Steady-state levels of protein *vs* the rate of synthesis of miRNA, *V*_SMIRNA_, illustrated in the presence of intermediate (n = 4 in Fig. S5A) or high degree of oligomerization (n = 12 in Fig. S5B) indicate that, with an intermediate level of oligomerization, the presence of miRNA is required to elicit the occurrence of sustained oscillations (Fig. S5A). However, with high levels of oligomerization, miRNA is not required to promote sustained oscillatory behavior (Fig. S5B).

Furthermore, the dynamical behavior of the model represented in the parameter plane defined by the degree of oligomerization between repressor molecules (n) and the rate of synthesis of miRNA (*V*_SMIRNA_) points out that a high degree of oligomerization is not needed to ensure sustained oscillations if an adequate level of miRNA is present (see Fig. S5C).

Let us remind that a necessary condition to generate the occurrence of sustained oscillations in the original Goodwin model is the presence of a high degree of oligomerization (n), with the condition that n≥8 [73]. This latter condition can be criticized since it assumes that at least 8 molecules of repressors must bind in a complex to be able to repress the expression of the gene. Here the model shows that the addition of a miRNA in the Goodwin model relaxes this strong condition about the high degree of oligomerization. This result suggests important roles of miRNAs in the regulation of the molecular mechanisms leading to the occurrence of biological rhythms.

### Dynamics of the “Goodwin-like” oscillator embedded in a ceRNA network

**Figure 9:**
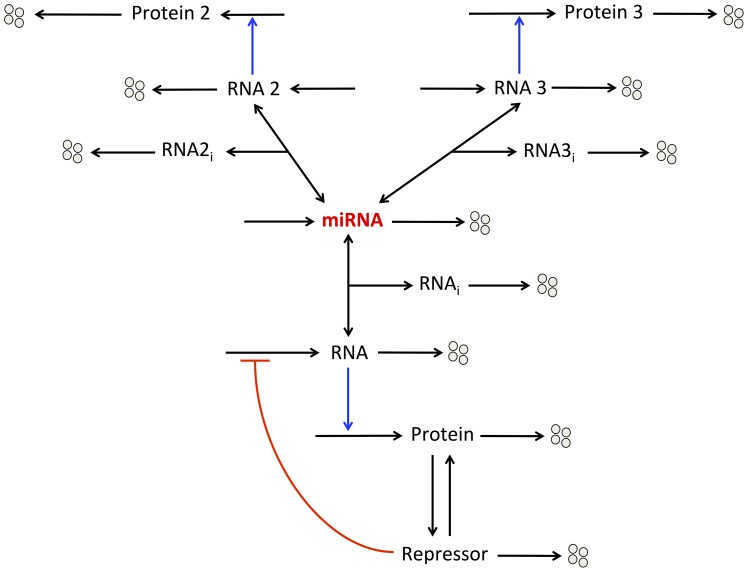
Wiring diagram of a “Goodwin-like” oscillator embedded in a minimal ceRNA network. One messenger RNA (RNA) ensures the synthesis of a protein, which can be converted into a repressor. This repressor down-regulates the synthesis of the RNA. By forming an inhibitory complex (RNA_i_) with the RNA, the miRNA reduces the level of messenger RNA. We hypothesize that the same miRNA also binds to other messenger RNAs (ceRNAs): RNAs 2 and 3. Those RNAs encode their respective proteins: Proteins 2 and 3.

Since most messenger RNAs in mammals can be a potential target for miRNA regulation [74], it seems natural to study the potential dynamical behavior of a “Goodwin-like” oscillator embedded in a ceRNA network. Indeed, we can reasonably assume that a miRNA, which binds to a messenger RNA involved in a negative feedback loop generating sustained oscillatory behavior, might also possess other messenger RNA targets, which are not directly involved in the core of the negative feedback loop. Thus, we further extend the “Goodwin-like” oscillator model, which already includes a miRNA, by the addition of two ceRNAs: RNAs 2 and 3 (see wiring diagram in Fig. 9). The latter ceRNAs can also bind to the miRNA, which prevent them to encode their respective proteins.

**Figure 10:**
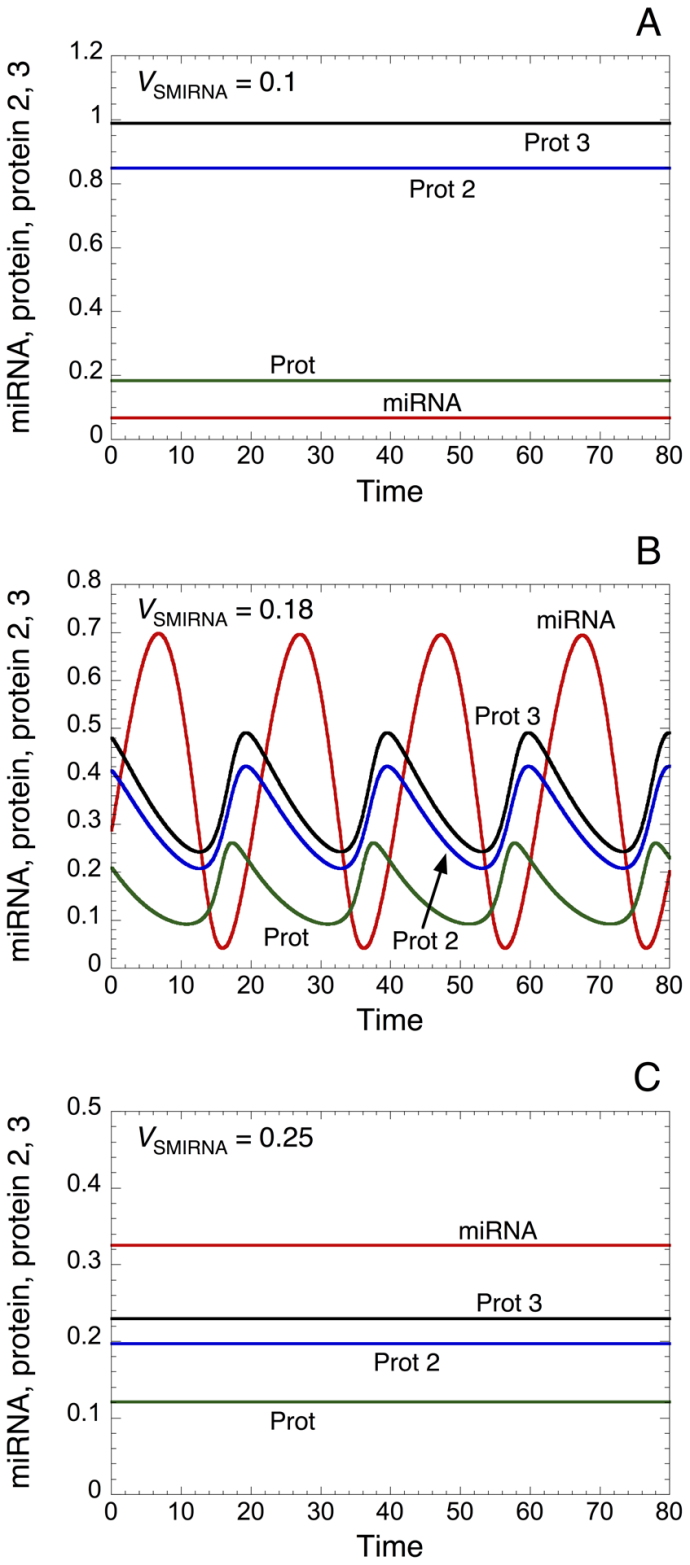
Propagation of oscillatory behavior within a ceRNA network. Time evolution of protein, protein 2, protein 3 and miRNA is illustrated in the presence of low (*V*_SMIRNA_ = 0.1), intermediate (*V*_SMIRNA_ = 0.18) and high (*V*_SMIRNA_ = 0.25) level of miRNA in panels A, B, and C, respectively. An appropriate level of miRNA creates a proper time-delay that favors the occurrence of sustained oscillations (see panel B). An elevated level of miRNA strongly represses the level of the different proteins, which abolishes the occurrence of sustained oscillatory behavior (panel C). Even if proteins 2 and 3 are not directly involved in the core of the oscillator, the model predicts that they can oscillate in phase with the protein directly involved in the negative feedback loop of the oscillator. Parameter values are as in Table 2 with n = 4.

Time evolution of miRNA, protein, and proteins 2 and 3 in the presence of low (*V*_SMIRNA_ = 0.1 in Fig. 10A), intermediate (*V*_SMIRNA_ = 0.18 in Fig. 10B), or large levels of miRNA (*V*_SMIRNA_ = 0.25 in Fig. 10C) indicates that intermediate levels of miRNA are required to elicit sustained oscillatory behavior (see also Fig. S5A). Steady-state levels of protein (Fig. S6A), protein 2 (Fig. S6B), protein 3 (Fig. S6C) and miRNA (Fig. S6D) towards the rate of synthesis of miRNA (*V*_SMIRNA_) further confirm this result by showing that the domain of sustained oscillatory behavior only occurs for intermediate levels of miRNA.

Interestingly, the model predicts that the levels of proteins 2 and 3, which are not directly involved in the core of the oscillator, can oscillate in phase with the protein directly involved in the negative feedback loop (see Fig. 10B). In the framework of biological rhythms, this result highlights the potential role of miRNAs as key molecular elements to extend the oscillatory behavior of proteins to proteins that are not directly involved in the core of the biological oscillator, but which are linked to the oscillator through a ceRNA network.

Simulations with the stochastic version of the “Goodwin-like” oscillator embedded in a ceRNA network (see Table 4 in Methods) indicate that sustained oscillations persist even with high molecular noise (see time evolution of miRNA, Prot, Prot2 and Prot3 in Fig. S7). As a result of random fluctuations near the two Hopf bifurcation points delimiting the oscillatory domain, the model shows that stochastic oscillations can occur even if the corresponding deterministic simulation predicts a stable steady state (compare Fig. S7F with Fig. 10C).

**Table 4:** Stochastic version of the “Goodwin-like” oscillator embedded in a ceRNA network (see scheme in Fig. 9).

Reaction number	Reaction	Propensity of reaction
1	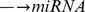	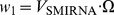
2	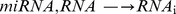	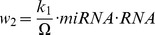
3	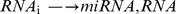	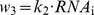
4	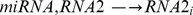	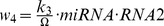
5	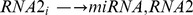	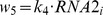
6	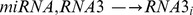	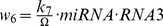
7	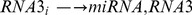	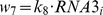
8	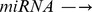	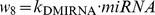
9	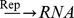	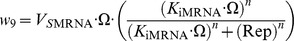
10	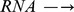	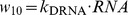
11	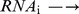	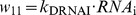
12	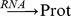	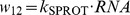
13	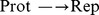	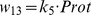
14	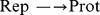	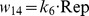
15	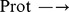	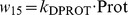
16	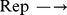	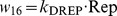
17	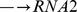	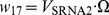
18	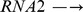	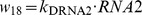
19	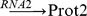	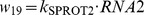
20	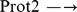	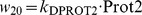
21	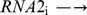	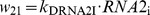
22	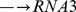	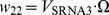
23	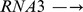	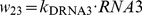
24	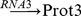	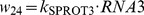
25	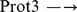	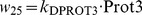
26	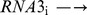	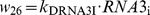

Furthermore, simulations of the “Goodwin-like” oscillator embedded in a ceRNA network within a heterogeneous cell population suggest that the oscillator is quite robust against random fluctuations of parameter values (see Fig. S8). Indeed, within the oscillatory domain (see Fig. 10B as well as Fig. S8D where *V*_SMIRNA_ = 0.18), a large proportion of cells (60%) still oscillate even with 30% of random variation on parameters. Near the two Hopf bifurcation points, the model predicts that some cells can enter into an oscillatory regime if sufficient random variation on parameters is present (see Figs. S8C, S8E).

## Discussion

miRNAs can greatly influence the level of protein expression and are also important to confer robustness to biological processes [3], [4], [7], [8], [75], [76]. Indeed, miRNAs and their target genes are often embedded in feedback and feed-forward regulatory motifs, which may improve the robustness of protein expression towards molecular noise [10], [41]. During the development of an organism, it was shown that a miRNA-mediated incoherent feed-forward loop could represent a filter that prevents the propagation of deleterious fluctuations in gene expression [77]. Moreover, a combined experimental and theoretical study also indicates that miRNAs can induce thresholds in protein expression [6]. Thresholds in protein synthesis can be also achieved by the presence of oligomerization of TF or amongst repressors for the regulation of gene expression [29], [78]. Such oligomerization mechanism could represent a way to control genetic noise [12], [79].

Here, we propose a minimal model for protein expression, which does not rest on any feedback and/or feed-forward regulatory motif between a gene and its miRNA. The model supports the view that oligomerization of TF as well as miRNA can induce the occurrence of thresholds in protein expression (see Fig. 1 and [6]). By resorting to stochastic simulations, we show that even small stochastic fluctuations in the copy number of messenger RNA have a great impact on protein output (Fig. 2). The minimal model already accounts for the fact that low levels of miRNA are sufficient to generate a robust buffer towards the undesirable stochastic expression of protein in the absence of TF (Fig. 3A, 3B as well as Fig. S1). Moreover, the model shows that high levels of miRNA could really act as a robust repressor of protein expression, even in the presence of TF eliciting RNA synthesis (Fig. 3E, 3F). Similarly, the model indicates that the presence of oligomerization of TF also ensures a robust buffer towards undesirable stochastic expression of protein (Fig. S1). By means of a deterministic model for a heterogeneous cell population, where every parameter can vary in a random manner within each cell in the population, we further show that the presence of miRNA also defines a robust buffer against undesirable protein synthesis (Fig. 4).

Most messenger RNAs are conserved target of miRNAs [74]. If several messenger RNAs bind to the same miRNA, each of these messenger RNAs represents a ceRNA for each other. Because ceRNAs are linked together through their common miRNA, their patterns of expression are positively correlated and they can form a large ceRNA regulatory network [31], [33]. Such ceRNA network might be of great importance in many physiological functions [31], such as in cell differentiation [80], or can play a key role in physiological disorders, such as in cell transformation and cancer development [56]–[58], [81].

To account for the qualitative dynamics of a minimal ceRNA network, we extend the model for protein expression by including two messenger RNAs: RNAs 2 and 3, which can bind to the same miRNA (Fig. 5). While the synthesis of those RNAs is not directly regulated by TF (the latter only controls the synthesis of RNA 1), the model shows that TF can control the level of proteins 2 and 3. This result supports previous observations showing that TF and ceRNA networks could be tightly intertwined in physiological as well as pathological conditions [32], [33]. Moreover, we show that the threshold in the synthesis of protein 1 generated by the oligomerization of TF can be transmitted to the expression profiles of proteins 2 and 3 (Fig. 6). The model further predicts that the propagation of such thresholds in protein synthesis may confer a robust buffer for the expression of all proteins involved in this network (Fig. 7). The model also indicates that the propagation of such thresholds in gene expression may only occur if the levels of the different ceRNAs are not too large as compared to the level of miRNA (Fig. S2). One could suggest that the propagation of thresholds in protein synthesis within a ceRNA network might represent a mechanism to limit the noise propagation as observed in gene networks [82].

Furthermore, an increasing amount of evidences illustrated the important roles of miRNAs in the control of the expression of proteins involved in negative feedback circuits regulating the occurrence of biological rhythms [83], [84]. These negative regulatory circuits allow the occurrence of biological rhythms such as the circadian clock [36], [68], [84], the calcium oscillations [35], the NFκB oscillations [62], [85], or the cell cycle [65].

By using a “Goodwin-like” model (see [37], [38] and Fig. 8), which may represent a generic biological oscillator, we assessed the effect of miRNA on the dynamics of such oscillator. We show that the addition of a miRNA regulating the level of the messenger RNA can act as a source of time-delay that favors the occurrence of oscillations of large amplitude (see Fig. 8). The latter result is supported by a recent experimental study, which showed that miRNAs are required to generate a time delay in the circadian clock oscillator [84]. Moreover, the model indicates that, in the presence of miRNA, the oscillator becomes less dependent from the high degree of oligomerization for the repression of the gene to ensure sustained oscillatory behavior (Figs. 8 and S5). This latter prediction suggests a key role of miRNAs for the occurrence of sustained oscillations, which may be of great importance for the control of biological rhythms [86].

Finally, we consider the potential dynamics of a “Goodwin-like” oscillator embedded in a ceRNA network (Fig. 9). While proteins 2 and 3 encoded respectively by RNAs 2 and 3 (ceRNAs) are not directly involved in the core of the oscillator, the model shows that, under intermediate levels of miRNA, they can oscillate in phase with the protein directly involved in the negative feedback loop (see Figs. 10 and S6). An extrapolation of this result could be that: if one ceRNA is involved in the core of a negative feedback loop and under adequate levels of its regulating miRNA, oscillatory behavior can be transmitted to every messenger RNA (and proteins) involved in this ceRNA network. In the framework of biological rhythms, this might extend considerably the oscillatory behavior of numerous proteins related, through the ceRNA network, but not directly involved in the core of the molecular mechanism generating the oscillations.

In summary, we developed a minimal model to account for the dynamics of protein expression regulated by oligomerization of TF and by miRNA. The model shows that both oligomerization and miRNA can induce thresholds in protein synthesis, which increase the robustness of gene expression towards stochastic fluctuations at a single cell level as well as towards random variation of parameter values. The latter variation may represent the variation that arises between cells in a population. By extending the model with two messenger RNAs (ceRNAs) binding to the same miRNA, we show that TF can modulate the level of the different proteins formed by this minimal ceRNA network, even if TF only promote the transcription of one gene in the network. This result supports previous observations showing that, through ceRNA network, TF regulatory potency could be much larger than expected [33]. In that framework, we further show that the robustness of gene expression elicited by the oligomerization of TF can be propagated to the profiles of expression of the other genes formed by the network. This result might have great implications for the robustness of the dynamics of gene expression in ceRNA networks. Moreover, we proposed a minimal “Goodwin-oscillator” model, which includes a miRNA. We show that the presence of miRNA in this oscillator creates a time-delay, which relaxes the strong requirement of high oligomerization between repressor molecules for the occurrence of sustained oscillations. Finally, the addition of ceRNAs in the latter oscillator indicates that the proteins formed by such ceRNA network can oscillate in phase with the protein directly involved in the negative feedback loop. This might greatly increase the oscillatory capability of the numerous proteins that may be formed by a ceRNA network. Thus, the model suggests that miRNAs can be seen as vectors allowing the propagation of robustness in protein expression as well as oscillatory dynamics within ceRNA networks.

## Methods

The different models proposed are described by a set of kinetic equations (see sections 1 to 4 here below) representing the time evolution of the concentration of the main variables driving the dynamics of protein expression. The different variables of the model are defined in Table 1, while the description of the parameters, together with their numerical values used in the simulations, are found in Table 2. The stochastic version of the minimal model for protein expression (see Fig. 1A) is presented in Table 3, while the stochastic version of the “Goodwin-like” oscillator embedded in a ceRNA network (see scheme in Fig. 9) is presented in Table 4. Both stochastic models consist of a set of reactions, which are directly related to the deterministic kinetic reactions and is simulated with the Gillespie algorithm [87].

### Kinetic equations of the model

All variables of the different models rest mainly on mass-action kinetics. The oligomerization of transcription factors (TF) for the control of messenger RNA synthesis is modeled with a Hill function, which includes the coefficient of cooperativity, n [43].

#### 1. Minimal model: one messenger RNA regulated by a miRNA (see scheme in Fig. 1A)



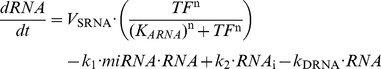
(1)

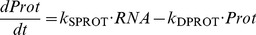
(2)

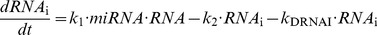
(3)

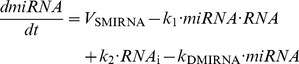
(4)


#### 2. Minimal model: 3 ceRNAs regulated by the same miRNA (see scheme in Fig. 5)

This model is described by the Eqs. [1] to [3] representing the time evolution of RNA, Prot and RNA_i_. The Eq. [4] representing the time evolution of miRNA is replaced by Eq. [4′] to include the terms of association and dissociation between miRNA and RNA2 as well as between miRNA and RNA3. Moreover, the Eqs. [5] to [10] describe the time evolution of RNA2, Prot2, RNA2_i_, RNA3, Prot3, and RNA3_i_, respectively.
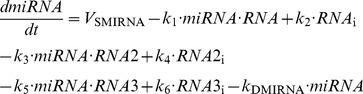
(4′)

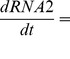
(5)

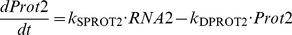
(6)

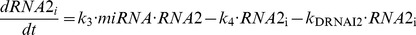
(7)

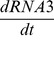
(8)

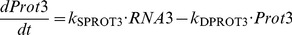
(9)

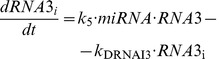
(10)


#### 3. “Goodwin-like” oscillator including a miRNA (see scheme in Fig. 8A)



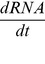
(11)

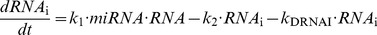
(12)

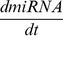
(13)

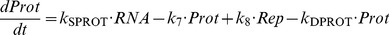
(14)

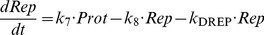
(15)


#### 4. “Goodwin-like” oscillator with a miRNA regulating 3 ceRNAs (see scheme in Fig. 9)

This model is described by Eqs. [11] to [15], where Eq. [13] representing the time evolution of miRNA is replaced by Eq. [4′] to include the terms of association and dissociation between miRNA and RNA2 as well as between miRNA and RNA3. Furthermore, Eqs. [5] to [10] representing the time evolution of RNA2, Prot2, RNA2_i_, RNA3, Prot3, and RNA3_i_ are also added to the model.

## Supporting Information

Figure S1 **Molecular noise in protein synthesis buffered by oligomerization of TF and by miRNA regulation of messenger RNA.** Maximum number of protein molecules *vs* TF are shown in the absence (*V*_SMIRNA_ = 0 in A, C) or presence of miRNA (*V*_SMIRNA_ = 0.01 in B, D), as well as in the absence (*n* = 1 in A, B) or presence of oligomerization of TF (*n* = 4 in C, D). Red lines indicate the arbitrary threshold of protein needed to ensure a cellular response. The presence of oligomerization of TF and miRNA induce an abrupt threshold in protein expression as a function of TF, below which protein is robustly repressed. 100 stochastic cells are considered for each condition. Parameter values are as in Table 2.
(TIFF)

Figure S2 **A ceRNA can act as a miRNA sponge.** Steady-state levels of protein 2 as a function of TF are shown with moderate (*V*_SMIRNA_ = 0.1 in A) and with high levels of miRNA (*V*_SMIRNA_ = 1 in B). For each case, different rates of synthesis of RNA 3 are considered: *V*_SRNA3_ = 0, 0.05, 0.1, and 0.25. As a result of common miRNA regulation, the level of TF (which only directly regulates protein 1 expression) can control the level of protein 2 (see also conditions of Fig. 7D). (A) An increase in the level of RNA 3 sponges the available miRNA, which prevents the miRNA from controlling the level of protein 2. (B) If a high level of miRNA is present, the level of RNA 3 is not large enough to sponge the miRNA. A significant amount of miRNA is still available to regulate the level of protein 2. Other parameter values can be found in Table 2.
(TIFF)

Figure S3 **Temporal dynamics of the minimal ceRNA network for different kinetics of transcription and translation of gene 3 (see scheme in** **Fig. 5** **).** Time evolution of RNA, RNA2, RNA3, Prot, Prot2, Prot3 and miRNA is illustrated with the default set of parameter values in panel A (see also conditions in Fig. 7B, D, F); with fast (set 2 in panel B) and slow kinetics of transcription and translation of gene 3 (set 3 in panel C). In set 2: *V*_SRNA3_ = 0.2, *k*_DRNA3_ = 1.3, *k*_SPROT3_ = 50, *k*_DPROT3_ = 2, *k*_DRNAI3_ = 1.6, *k*_5_ = 100; while in set 3: *V*_SRNA3_ = 0.002, *k*_DRNA3_ = 0.01, *k*_SPROT3_ = 0.5, *k*_DPROT3_ = 0.02, *k*_DRNAI3_ = 0.01, *k*_5_ = 1. Other parameter values are as in Fig. 7B, D, F.
(TIFF)

Figure S4 **Propagation of robustness in protein synthesis within a ceRNA network for different kinetics of expression of gene 3.** Levels of protein 2 (A, B) and protein 3 (C, D) are represented in a heterogeneous cell population (100 cells) as a function of the percentage of random variation around the default value for each parameter of the model. Simulations are performed with the set 2 (A, C) and the set 3 (B, D) of parameter values (see legend of Fig. S3). Red lines define the arbitrary threshold of protein needed to generate a cellular response. Other parameter values are as in Fig. S3.
(TIFF)

Figure S5 **miRNA as a source of time delay in a “Goodwin-like” oscillator.** Steady-state levels of protein as a function of the rate of synthesis of miRNA, *V*_SMIRNA_, are represented for an intermediate (*n* = 4 in A) and for a high degree of oligomerization between repressor molecules (*n* = 12 in B). Solid curves: stable steady states or envelope, i.e. minima and maxima, of the sustained oscillations; dashed curves: unstable states. (C) Dynamical behavior of the model illustrated in a two-parameter bifurcation diagram defined by the degree of oligomerization, *n*, and the rate of synthesis of miRNA, *V*_SMIRNA_. Parameter values are as in Table 2.
(TIFF)

Figure S6 **Domain of oscillatory behavior of a “Goodwin-like” oscillator embedded in a minimal ceRNA network.** Steady-state levels of protein, protein 2, protein 3 and miRNA as a function of the rate of synthesis of miRNA, *V*_SMIRNA_, are shown in panels A to D, respectively. Solid curves: stable steady states or envelope of the sustained oscillations; dashed curves: unstable states. Intermediate levels of miRNA are needed to promote the occurrence of sustained oscillations within the ceRNA network. Parameter values are as in Fig. 10.
(TIFF)

Figure S7 **Effect of stochastic fluctuations on the dynamics of the “Goodwin-like” oscillator embedded in a ceRNA network (see scheme in** **Fig. 9** **).** Stochastic time evolution of miRNA, protein, proteins 2 and 3 in the presence of low (*V*_SMIRNA_ = 0.1 in A, B), intermediate (*V*_SMIRNA_ = 0.18 in C, D) or high (*V*_SMIRNA_ = 0.25 in E, F) levels of miRNA. Simulations are performed in the presence of low (Ω = 10000 in A, C, E) or large (Ω = 1000 in B, D, F) molecular noise. The stochastic version of the “Goodwin-like” oscillator embedded in a ceRNA network is described in Table 4. Parameter values are as in Fig. 10.
(TIFF)

Figure S8 **Robustness of the “Goodwin-like” oscillator embedded in a ceRNA network within a heterogeneous cell population.** Proportion of oscillating and non-oscillating cells is plotted towards the percentage of random variation on every parameter of the model for increasing rates of synthesis of miRNA, *V*_SMIRNA_. From condition A to F, *V*_SMIRNA_ = 0.01, 0.05, 0.1, 0.18, 0.25, and 0.5, respectively. Default values for the parameters are as in Fig. 10.
(TIFF)
